# Long non-coding RNA-NEAT1, a sponge for miR-98-5p, promotes expression of oncogene HMGA2 in prostate cancer

**DOI:** 10.1042/BSR20190635

**Published:** 2019-09-24

**Authors:** Zhuifeng Guo, Chang He, Fan Yang, Liang Qin, Xuwei Lu, Jiawen Wu

**Affiliations:** Department of Urology, Shanghai Minhang Hospital, Fudan University, Shanghai 201199, People’s Republic of China

**Keywords:** HMGA2, lncRNA NEAT1, miR-98-5p, prostate cancer

## Abstract

Increasing evidence demonstrated that noncoding RNAs (lncRNA, miRNA) play important roles in the cancer development. LncRNA NEAT1 functions as an oncogene in many cancers. However, the roles of NEAT1 in prostate cancer (PCa) remain largely unknown. In the present study, we aim to explore the molecular mechanism of NEAT1 in the development of PCa. We detected the expression levels of NEAT1 in a total of 16 benign prostatic hyperplasia tissues (BPH), 30 matched adjacent healthy control (HC) tissues and 30 PCa tissues, as well as PCa cell lines PC-3, DU-145, LNCaP and normal prostate epithelial cell line RWPE-1. The results showed that NEAT1 was significantly up-regulated in PCa tissues and PCa cell lines. Knockdown of NEAT1 can largely inhibit DU-145 and PC-3 cell growth and invasion. Bioinformatics analysis predicted NEAT1 has the binding site of miR-98-5p which can bind to the 3′UTR of HMGA2. And the expression level of NEAT1 has a positive correlation with HMAG2, while negative correlation with miR-98-5p in PCa cells. In addition, luciference assay and RNA immunoprecipitation (RIP) assay confirmed that NEAT1 can function as a competing endogenous RNA (ceRNA) by sponging miR-98-5p to active HMGA2. Moreover, silencing of HMGA2 can decrease the proliferation ability of PCa cells. Taken together, NEAT1/miR-98-5p/HMGA2 pathway is involved in the development and progression of PCa. NEAT1 could be recommended as a prognostic biomarker and inhibition of NEAT1 expression may be a promising strategy for PCa therapy.

## Introduction

Prostate cancer (PCa) is the most common malignancy and the second leading cause of cancer death among men worldwide [1]. The occurrence and development of PCa is a multi-step process involving in the deregulation of many oncogenes and tumor suppressor genes. Despite great progresses have been made to understand the complicated pathogenesis of PCa and to improve its diagnosis and treatment efficiency, the long-term survival remains dismal and PCa still remains a severe disease [2,3]. Therefore, it is urgent to explore the molecular mechanisms underlying PCa and develop promising diagnostic and prognostic biomarkers for cancer diagnosis and treatment.

LncRNAs, a class of non-coding RNAs with the length ranging from 200 nucleotides to almost 100 kilobases, play important roles in cancer progression and development [4]. Accumulating evidences showed that lncRNAs are involved in several cell biological processes including cell growth, differentiation, apoptosis, angiogenesis, migration, invasion, metastasis and cell-cycle progression [5,6]. Recent studies demonstrated that lncRNAs function as miRNA sponges and therefore exert their oncogenic or tumor suppressive effect [7], which suggesting the pivotal roles of lncRNAs in the development and progression of PCa.

LncRNA NEAT1 was found to function as an oncogene in different kinds of cancer [8,9]. It has been reported to regulate miRNAs activity in a series of cancers. Among these miRNAs, NEAT1 is found to act as a competing endogenous lncRNA for miR-98-5p in non-small-cell lung cancer [10]. However, the role of NEAT1 in PCa and whether NEAT1 is able to regulate miR-98-5p to promote PCa progression is still unclear.

In the present study, we demonstrated that NEAT1 was significantly up-regulated in PCa tissues and cell lines, and associated with higher Gleason score and advanced TNM stage. Knock down of NEAT1 can suppress PCa cells proliferation and invasion *in vitro*. Furthermore, we also found that HMGA2 was a direct target gene of miR-98-5p. NEAT1 regulated HMGA2 expression by acting as a competing endogenous RNA (ceRNA) in PCa cells. Taken together, the present study may demonstrate that NEAT1/miR-98-5p/HMGA2 pathway is involved in the growth and invasion of PCa cells *in vitro*. NEAT1 could be regarded as a diagnostic and prognostic biomarker for PCa diagnosis and treatment.

## Materials and methods

### Tissues samples

All benign prostatic hyperplasia (BPH), PCa and matched adjacent healthy control (HC) tissues were obtained from the Department of urology, Shanghai Minhang Hospital between January 2015 and December 2017. All samples were confirmed by pathological method and stored in liquid nitrogen in accordance with the World Medical Association Declaration of Helsinki. All patients have accepted consent for the use of all samples. Study protocol for the present study and informed consent documents were reviewed and approved by the Ethics Committee of Shanghai Minhang Hospital, Fudan University. All the participants provided written informed consent during their hospitalization.

### Cell culture

The human PCa cell lines LNCaP, PC-3, DU-145 and normal prostate epithelial cell line RWPE-1 were obtained from the American Type Culture Collection (ATCC). The four cell lines are all cultured in RPMI1640 medium supplemented with 10% fetal bovine serum (FBS, Gibco) at 37°C in humidified air containing 5% of CO_2_ according to the manufacturer’s instructions.

### Vectors and transfection

The wild-type (WT) or MT 3′UTR of HMGA2 containing the predicted potential miR-98-5p-binding sites were cloned into the luciference reporter vector p-Luc. siRNA of HMGA2 and NEAT1, miR-98-5p mimics were purchased from GenePharma (China). All cell transfections were performed by using the Lipofectamine 2000 (Invitrogen, U.S.A.) following to the manufacturer’s instructions.

### Cell proliferation assay

Cell viability was determined by Cell Counting Kit 8 assay (CCK8) according to the manufacturer’s instructions. For the colony formation assay, 800–1000 cells were seeded in 12-well plate and maintained in complete medium for 2 weeks. Then washed in PBS and fixed with methanol and stained with 0.1% crystal violet for 15–20 min. Then the number of clones were counted and taken pictures using a microscope.

### Cell invasion assay

Transwell assay was performed to determine cell invasion ability. Fifty microliter diluted Matrigel™ (1:8, Sigma–Aldrich, U.S.A.) was added to the upper chamber of the transwell. 5 × 10^4^ cells were resuspended in 100 μl serum-free RPMI1640 medium, and seeded in the upper chamber in 24-well plates (Corning, U.S.A.). Five hundred microliter completed medium was added in lower chamber at 37°C incubator. After 24-h incubation, the cells on the upper side of membrane were scraped off and fixed lower side cells with 4% PFA and then stained with 0.1% Crystal Violet for 15–20 min.

### Dual luciferase reporter assay

DU-145 and PC-3 cells were cultured in 24-well plates with 80% cell density and co-transfected with miR-98-5p mimics or NEAT1 siRNA and luciferase reporter vectors (containing HMGA2 3′UTR WT or HMGA2 3′UTR MT) and *Renilla* vectors using Lipofectamine 2000 (Invitron). After 24 h, the luciferase activities were measured by using the Dual-Luciferase Reporter Assay System (Promega, U.S.A.) following to the manufacturer’s instructions.

### RNA isolation and qRT-PCR

Total RNA was extracted using TRIzol Reagent (Invitrogen, U.S.A.). cDNA was synthesized using PrimeScript™ RT Master Mix Kit (TaKaRa, RR037A) or TIANGEN^®^ miRcute Kit (KR211).

Quantitative RT-PCR was measured using SYBR^®^ Premix Ex Taq™ (TaKaRa, RR420A) or TIANGEN^®^ miRcute Plus miRNA qPCR Kit (FP411). The relative expression of NEAT1, HMGA2 and miR-98-5p were calculated by the 2^−ΔΔ^*^C^*_t_ method.

### Western blot assay

For WB, all cells were collected and lysed in RIPA lysis buffer. Proteins were resolved on SDS/PAGE gels and transferred proteins to negative controls (NC) membranes, then incubated membrane in blocking buffer for 1 h and incubated primary antibody overnight. Next day, washed membrane in PBS for three times and incubated second antibody for 1 h. The primary antibodies used were: HMGA2 (Abcam, ab97276), β-actin (Abcam, ab8226).

### RNA immunoprecipitation

RNA-IP was performed using a kit from Active Motif (Carlsbad, CA, U.S.A.) following the manufacturer’s protocol. PC-3 cells were lysed in RNA immunoprecipitation (RIP) lysis buffer and magnetic beads conjugated to human anti-Ago2 antibody (Millipore) or control IgG antibody. Then the samples were digested with proteinase K, and RNA was extracted from the beads using TRIzol. Then performed qRT-PCR analysis to measure the expression of the NEAT1 and miR-98-5p. The primers are available in the Table 1.

**Table 1:** Primer sequence

Name	Sequence (5′–3′)
NEAT1-F	CAGGGTGTCCTCCACCTTTA
NEAT1-R	AAACCAGCAGACCCCTTTTT
miR-98-5p-F	GGGGTGAGGTAGTAAGTTGT
miR-98-5p-R	TGGGTGTCGTGGAGTC
HMGA2-F	CAGCAGCAAGAACCAACCG
HMGA2-R	TGTTGTGGCCATTTCCTAGGT
U6-F	ATTGGAACGATACAGAGAAGATT
U6-R	GGAACGCTTCACGAATTT
GAPDH-F	GACTCATGACCACAGTCCATGC
GAPDH-R	AGAGGCAGGGATGATGTTCTG

### Statistical analyses

All the statistical analyses were performed using SPSS 17.0. Data were indicated as mean ± SD from three independent experiments. To compare the differences among two or multiple groups, paired Student’s *t* test or one-way ANOVA were used. It was considered significant when *P*-value <0.05.

## Results

### NEAT1 is up-regulated in PCa cells and tissues

**Figure 1:**
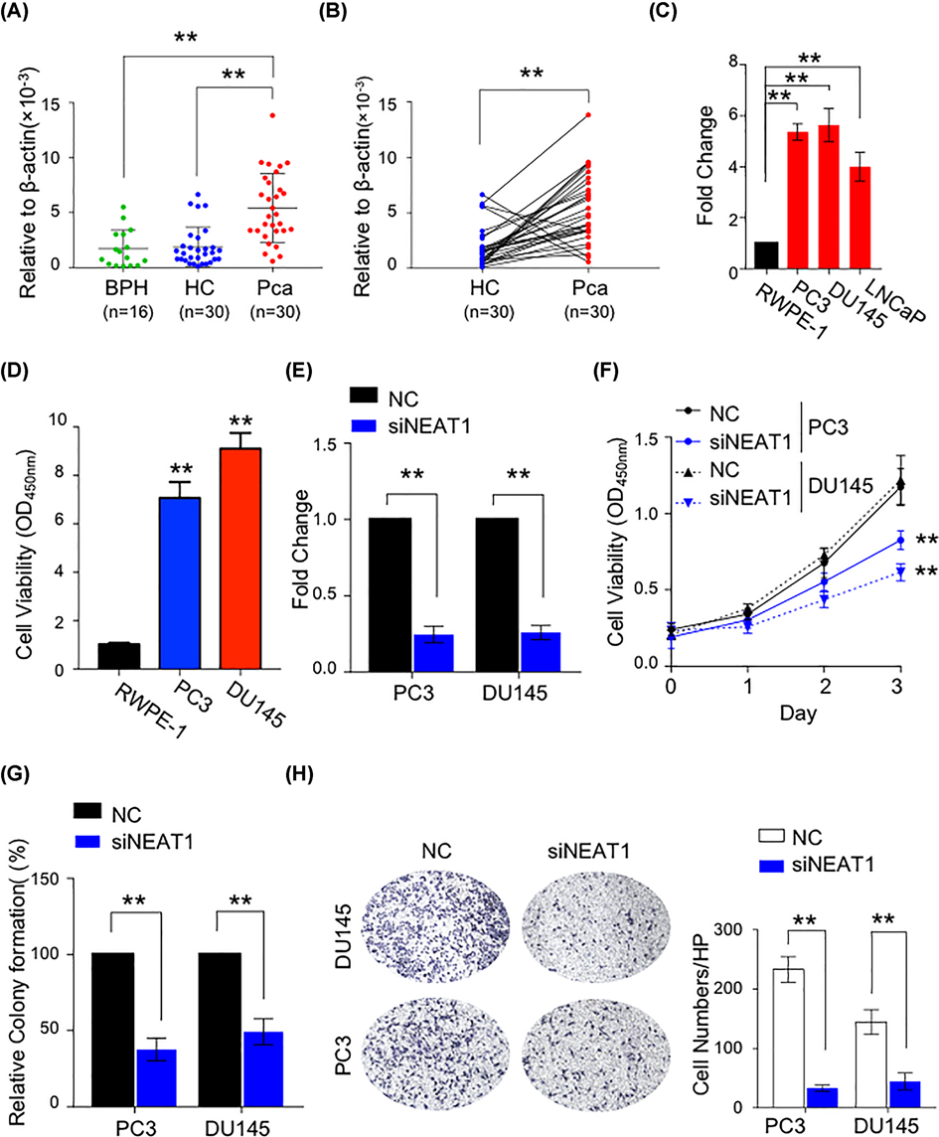
Overexpression of NEAT1 promote cell proliferation and invasion in PCa cells (**A,B**) Relative expression level of NEAT1 in BPH, PCa and matched adjacent HC tissues. **P<0.01. (**C**) The expression level of NEAT1 in normal prostate epithelial cell line RWPE-1 and PCa cell lines PC-3, DU-145 and LnCaP cells. **P<0.01, Mann–Whitney *U* test was used to compare PCa cells and normal cells. (**D**) Cell proliferation ability was determined by CCK8 assay in RWPE-1, PC3 and DU145 cell lines. **P<0.01. (**E**) The NEAT1 expression level with or without NEAT1 siRNA transfection in PC-3 and DU-145 cell lines. **P<0.01. (**F**) Cell viability was determined of control and NEAT1 knockdown groups in different time points in PC-3 and DU-145 cell lines. **P<0.01. (**G**) Relative colony formation percent after knockdown of NEAT1 in two PCa cell lines. **P<0.01. (**H**) The transwell assay was performed to evaluate the cell ability of invasion with or without NEAT1 siRNA. The picture of invasion cells in two groups (left side) and the relative invasion cell number in two groups (right side). **P<0.01.

To investigate the role of NEAT1 in PCa, we first measured NEAT1 expression in BPH, PCa and matched adjacent HC tissues. Although NEAT1 level exhibits no obvious difference in BPH and HC tissues (*P*>0.05, Figure 1A), it is clearly showed that the expression level of NEAT1 is significantly up-regulated in PCa tissues, compared with BPH (*P*<0.0001) and HC tissues (*P*<0.0001, with average 6.42-fold increase compared with matched healthy tissues, Figure 1B). Consistently, the overexpression of NEAT1 in PCa is also validated in cell lines, as shown that NEAT1 is up-regulated in DU145, PC3 and LNCaP PCa cell lines compared with normal prostate epithelial cell line RWPE- 1 (Figure 1C). Then cell proliferation ability was determined by CCK8 assay in RWPE-1, PC3 and DU145 cell lines. It showed that the proliferation ability of PC3 and DU145, which have high NEAT1 expression level, was much stronger that normal prostate epithelial cell line RWPE-1 (Figure 1D). Taken together, these finding suggest that NEAT1 may play a key role in the progression of PCa.

### The overexpression of NEAT1 is correlated with advanced clinical stage of disease

We further investigated the clinical significance of NEAT1 expression in PCa tissues from a larger cohort (*n*=122). Based on NEAT1 expression in PCa tissues, the patients were divided into NEAT1 low and high-expressing group. The medium value of NEAT1 expression in cancer tissues was determined as cut-off value. As shown in Table 2, the higher level of NEAT1 is significantly correlated with advanced stage (*P*=0.0007) and higher Gleason score (*P*=0.0060), suggesting that overexpression of NEAT1 may contribute to malignant behaviors of PCa cells.

**Table 2:** Correlation of NEAT1 expression and clinicopathological variables in PCa

		NEAT 1			
		*n*=122	Low (*n*=61)	High (*n*=61)	*P*-value
Age	≥75	61	28	33	0.4689
	<75	61	33	28	
Smoking	Yes	6	4	2	0.6755
	No	116	57	59	
TNM staging	I	32	25	7	**0.0007
	II	42	19	23	
	III+IV	48	17	31	
Gleason score	≥8	70	27	43	**0.0060
	<8	52	34	18	

### Down-regulation of NEAT1 inhibit cell growth and invasion in PCa cells

Based upon above clinical data, we therefore performed several *in vitro* assays to show the biological relevance of NEAT1 in PCa. Since PC3 and DU145 had higher level of NEAT1 (Figure 1C) and high-proliferation ability (Figure 1D), we aimed to inspect the impact of reduced NEAT1 on biological phenotype of PCa cells. Accordingly, we treated those cells with siRNA mimics specific for NEAT1. As shown in Figure 1E, the NEAT1 level was significantly reduced by approximately 70%, suggesting that we successfully established NEAT1-knockdown PCa cells.

By using CCK-8 assay, we observed that the cell viability of NEAT1-knockdown cells was significantly inhibited compared with control cells (Figure 1F). Likewise, PC3 and DU145 cells with reduced NEAT1 expression showed impaired colony formation capability (Figure 1G), suggesting that high-expression level of NEAT1 is required for cell growth in PCa cells. Furthermore, we evaluated the invasion ability of control cell or NEAT1-knockdown PCa cells by using Transwell-Matrigel invasion assay. In line with our hypothesis, NEAT1 siRNA treatment significantly inhibited cell invasion in PC3 and DU145 cells (*P*<0.001, Figure 1H). Collectively, our data clearly showed NEAT1 contributes to cell growth and invasion in PCa cells.

### NEAT1 acts as a miR-98-5p sponge to up-regulate oncogene HMGA2 in PCa cells

Recent studies demonstrated that lncRNAs function as miRNA sponges and therefore exert their oncogenic or tumor suppressive effect. NEAT1, for instance, have been reported to regulate miRNAs activity in different kinds of cancer. Among these miRNAs, NEAT1 is found to act as a competing endogenous lncRNA for miR-98-5p in lung cancer. However, whether NEAT1 is able to regulate miR-98-5p in PCa remains unclear.

**Figure 2:**
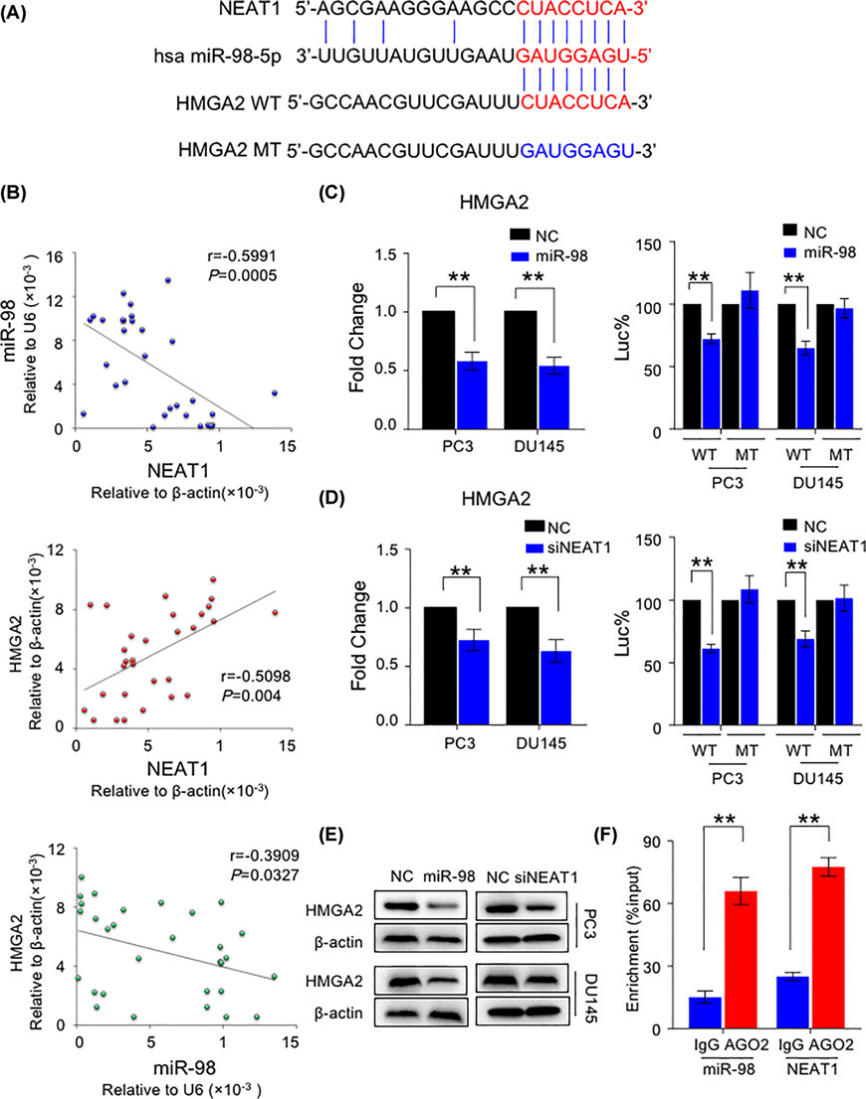
NEAT1 acts as a miR-98-5p sponge to up-regulate oncogene HMGA2 in PCa cells (**A**) The binding sites of miR-98-5p on WT NEAT1 (indicated in red), WT HMGA2 (indicated in red) and the mutant type HMGA2 (indicated in blue). (**B**) The relative expression level between miR-98-5p and NEAT1, HMGA2 and NEAT1, HMGA2 and miR-98-5p. (**C**) The HMGA2 mRNA expression level with control or miR-98-5p mimics overexpression in PC-9 and DU-145 cell lines (left side). **P<0.01. The relative luciference activity with control or miR-98-5p mimics overexpression in HMGA2 WT or MT groups in two cell lines (right side) **P<0.01. (**D**) The relative HMGA2 mRNA expression level with control and knockdown of NEAT1 in PC-9 and DU-145 cell lines (left side). **P<0.01. The relative luciference activity with control and knockdown of NEAT1 in HMGA2 WT or MT groups in two cell lines (right side) **P<0.01. (**E**) The HMGA2 protein expression level with control or miR-98-5p mimics overexpression in PC-9 and DU-145 cells. (**F**) RIP assay was performed to confirm the interaction between miR-98-5p and AGO2, NEAT1 and AGO2 in PC-9 cell lines. **P<0.01.

By utilizing microRNA.org algorithm (www.microRNA.org), we identified binding sites of miR-98-5p in NEAT1 (Figure 2A). Intriguingly, we also found HMGA2 as a novel potential target of miR-98-5p. HMGA2 is intensively reported as an oncogene to promote tumor progression in multiple carcinoma. We therefore hypothesized that NEAT1 may promote HMGA2 expression through inhibiting miR-98-5p activity in PCa. To confirm our assumption, we first analyzed the expression correlation of NEAT1, miR-98-5p and HMGA2. Consistently, the expression level of NEAT1 has a positive correlation with HMAG2, while negative correlation with miR-98-5p in PCa cells (Figure 2B), suggesting that NEAT1-miR-98-5p-HMGA2 regulatory axis may have biological relevance during the development of PCa.

To support the hypothesis that HMGA2 is a novel target of miR-98-5p, PC3 and DU145, cells were subjected to miR-98-5p mimics and negative control treatment. As expected, HMGA2 mRNA level was significantly reduced by miR-98-5p overexpression (Figure 2C). Furthermore, to confirm the direct interaction between miR-98-5p and HMGA2 *in vitro*, we cloned miR-98-5p-binding sites of HMGA2 into luciferase report plasmid to establish WT of HMGA2 3′-UTR report plasmids. Besides, we mutated miR-98-5p-binding region and constructed mutant 3′-UTR regions of HMGA2. PC-3 and DU-145 cells were transiently transfected with these constructs along with miR-98-5p mimics or NC. In line with our hypothesis, miR-98-5p overexpression remarkably inhibited luciferase activity of the reporter genes containing WT 3′-UTR regions of HMGA2, but no inhibitory effects were observed in mutated cell lines, suggesting that HMGA2 is a direct target of miR-98-5p in PCa cells (Figure 2C). And we also showed that the expression level of HMGA2 and miR-98-5p were negatively correlated in PCa tissues (Supplementary Figure S2C)*.*

We next treated PCa cells with NEAT1 siRNA or negative controls. Consistently, we found NEAT1 knockdown significantly suppress luciferase activity of the reporter genes containing 3′-UTR regions of HMGA2, as well as HMGA2 mRNA level, supporting that reduction in NEAT1 expression caused re-activated miR-98-5p and enhanced inhibitory effect on HMGA2 3′-UTR by miR-98-5p (Figure 2D). Furthermore, we also validated that knockdown of NEAT1 led to down-regulation of HMGA2 protein level (Figure 2E). To further confirm the physical interaction between NEAT1 and miR-98-5p, we performed RIP assay. As shown in Figure 2F, both miR-98-5p and NEAT1 are able to bind to Ago2 protein in PC3 cells. Together, we for the first time showed that NEAT1 functions as a miR-98-5p sponge to activate oncogene HMGA2 in PCa cells.

### HMGA2 contributes to cell proliferation and invasion in PCa cells

**Figure 3:**
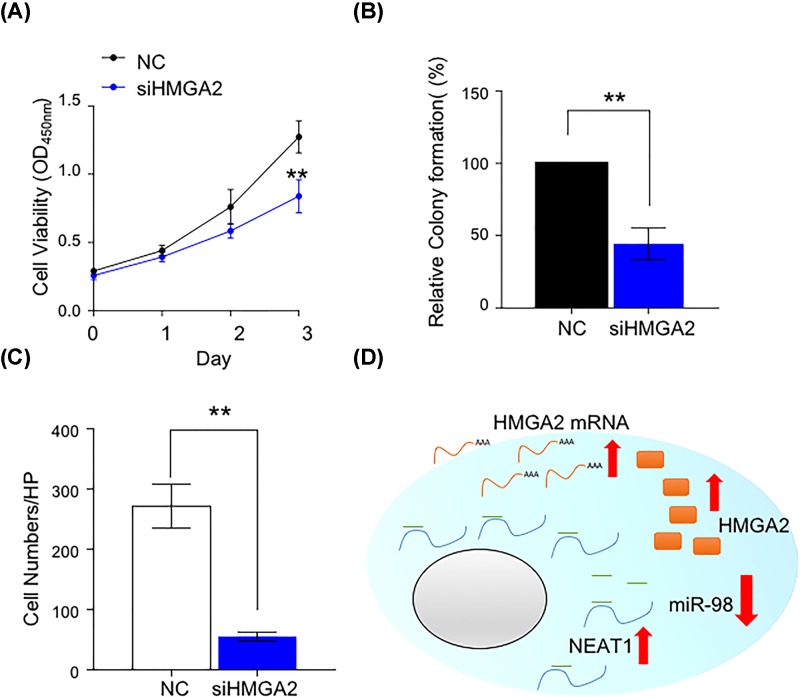
HMGA2 contributes to cell proliferation and invasion in PCa cells (**A**) CCK8 assay was performed to evaluate cell viability in control or knockdown of HMGA2 groups. **P<0.01. (**B**) Cell proliferation ability was detected in control or knockdown of HMGA2 groups by colony formation assay. **P<0.01. (**C**) Cell invasion ability was performed by transwell assay in control or knockdown of HMGA2 groups. **P<0.01. (**D**) Schematic diagram of mechanism of the present study. NEAT1 suppressed the expression of miR-98-5p by serving as a ceRNA of HMGA2 to induce the expression of HMGA2, and lead to the accumulation of HMGA2 to drive PCa growth and invasion.

Since NEAT1 is able to regulate HMGA2 expression in PCa cells, we then questioned whether HMGA2 in fact has oncogenic role in the development of PCa cells. We subsequently treated PC3 cells by using HMGA2 siRNA or NC. As shown in Figure 3A, the cell viability was remarkably suppressed by HMGA2 knockdown. In addition, HMGA2 inhibition led to the reduced colony formation (Figure 3B) and invasion ability (Figure 3C). Collectively, these findings confirmed that HMGA2 functions as oncogene in PCa cells.

## Discussion

There is growing evidence which showed that non-coding RNAs (lncRNA, miRNA, circRNA) are recognized as important gene regulators in PCa. Therefore, it is urgent to identify and elucidate the potential roles of lncRNAs or miRNAs involved in PCa.

In the present study, we found that the expression level of NEAT1 was significantly up-regulated in the PCa tissues and cell lines (Figure 1A–C), high expression of NEAT1 was positively associated with high-Gleason score and advanced TNM stage (Table 1). In the cell function assay, we showed that silencing the expression of NEAT1 can largely suppressed PCa cell growth and invasion *in vitro* (Figure 1D–G). Therefore, these findings revealed that lncRNA NEAT1 act as an oncogene in PCa. Moreover, we found that NEAT1 was negatively associated with miR-98-5p, which highly expressed in PCa tissues, whereas miR-506 and miR-193a-3p expression were no difference between HC and PCa tissues (Supplementary Figure S1A–C), meanwhile miR-98-5p were positively associated with HMGA2 (Figure 2B). In addition, HMGA2 was speculated as a target of miR-98-5p and NEAT1 (Figure 2A). Luciference assay verified that NEAT1 and HMGA2 3′UTR containing the binding site of miR-98-5p (Figure 2C,D). RIP assay also demonstrated that miR-98-5p and NEAT1 could interact with AGO2 (Figure 2F). Not unexpectedly, we found that overexpression of miR-98-5p can largely suppressed the protein expression of HMGA2 (Figure 2E). And we found that miR-98-5p was largely low expressed in PCa tissues, whereas HMGA2 was highly expressed in PCa tissues (Supplementary Figure S2A,B). And the expression level between miR-98-5p and HMGA2 were negatively correlated in PCa tissues (Supplementary Figure S2C). Taken together, we hypothesized that NEAT1/miR-98-5p/HMGA2 axis was involved in the development and progression of PCa *in vitro*.

Previous studies showed that lncRNA NEAT1 played vital roles in the development of tumors, such as lung cancer, hepatocellular carcinoma, colorectal cancer and squamous cell carcinoma [11–14]. For instance, Luo et al. [12] demonstrated that NEAT1 can promote colorectal cancer progression by competing binding miR-34a to induce the expression of SIRT1 through enhancing the Wnt/β- catenin signaling pathway. Yong et al. [15] showed that NEAT1 promotes cell proliferation and migration through sponging miR-506 in high-grade serous ovarian cancer. Xiong et al. [16] revealed that NEAT1 plays important roles in the progression and occurrence of lung adenocarcinoma through acting as a ceRNA to regulate miR-193a-3p. And Ling et al. [17] showed that NEAT1 contributes to the deterioration of HCC and provides a potential biomarker for the diagnosis and therapy of HCC. Inhibition of NEAT1 can suppress cancer cell proliferation, migration, invasion, cell cycle and metastasis. However, the role of NEAT1 in PCa is poorly understood.

In the present study, we revealed that the expression level of NEAT1 was highest in PCa tissues compared with BPH and normal tissues. Moreover, the expression of NEAT1 was positively associated with high-Gleason score and advanced TNM stage. All these results suggested that NEAT1 may play important roles in the pathogenesis and progression of PCa.

NEAT1 has been reported to regulate miRNAs activity in different kinds of cancer. Among these miRNAs, NEAT1 is found to act as a competing endogenous lncRNA for miR-98-5p in lung cancer [10]. However, whether NEAT1 is able to regulate miR-98-5p in PCa remains unclear. To investigate the target gene of NEAT1, we used bioinformatics analysis. Intriguingly, we not only found that miR-98-5p is a target of NEAT1, but also found HMGA2 as a novel potential target of miR-98-5p. We identified miR-98-5p as potential miRNA that links NEAT1 and HMGA2. HMGA2 is intensively reported as an oncogene to promote tumor progression in multiple carcinoma [18–21]. Therefore, we hypothesized that NEAT1 may promote HMGA2 expression by inhibiting miR-98 -5p activity in PCa.

To better explain the molecular mechanism of NEAT1/miR-98-5p/HMGA2 axis in the development of PCa, we first analyzed the expression correlation of NEAT1, miR-98-5p and HMGA2. Consistently, the expression level of NEAT1 has a positive correlation with HMAG2, while negative correlation with miR-98-5p in PCa cells (Figure 2B), suggesting that NEAT1-miR-98-5p-HMGA2 regulatory axis may have biological relevance during the development of PCa. Knock down of HMGA2 could largely decrease the growth and invasion ability of PCa cell lines.

In summary, the present study demonstrated that lncRNA NEAT1 could function as a ceRNA to promote HMGA2 expression by binding miR-98-5p. NEAT1/miR-98-5p/HMGA2 signaling pathway plays a critical role in the growth, proliferation, migration and invasion of PCa cells. Therefore, lncRNA NEAT1 might serve as a therapeutic target as well as a prognostic biomarker in PCa.

## Supporting information

**Supplementary Figure S1:** 

**Supplementary Figure S2:** 

## Abbreviations

BPH: benign prostatic hyperplasia
CCK8: Cell Counting Kit 8
ceRNA: competing endogenous RNA
HC: healthy control
NC: negative control
PCa: prostate cancer
RIP: RNA immunoprecipitation
WT: wild-type
