# Supplementary figures and images for: Long non-coding RNA-NEAT1, a sponge for miR-98-5p, promotes expression of oncogene HMGA2 in prostate cancer
Source: Biosci Rep. 2019 Sep 24;39(9):BSR20190635. doi: 10.1042/BSR20190635 (PMC6757183; doi:10.1042/BSR20190635)

Figure S1

A.

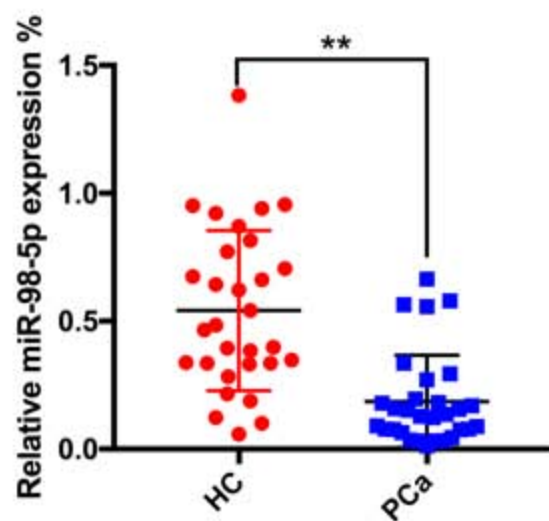

B.

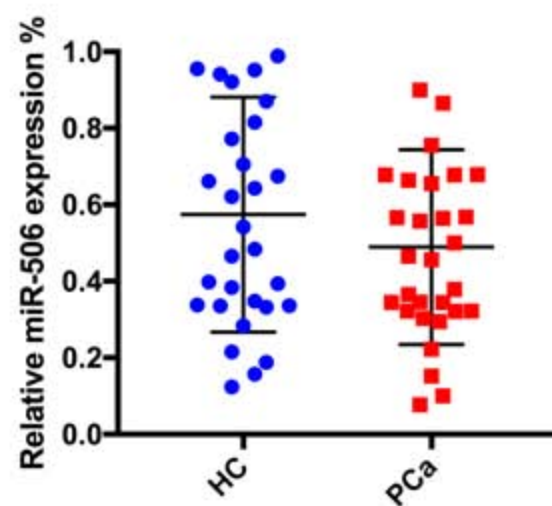

C.

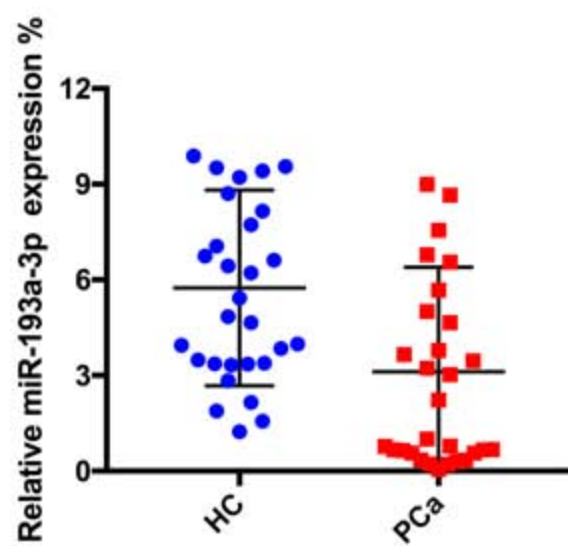

Figure S2

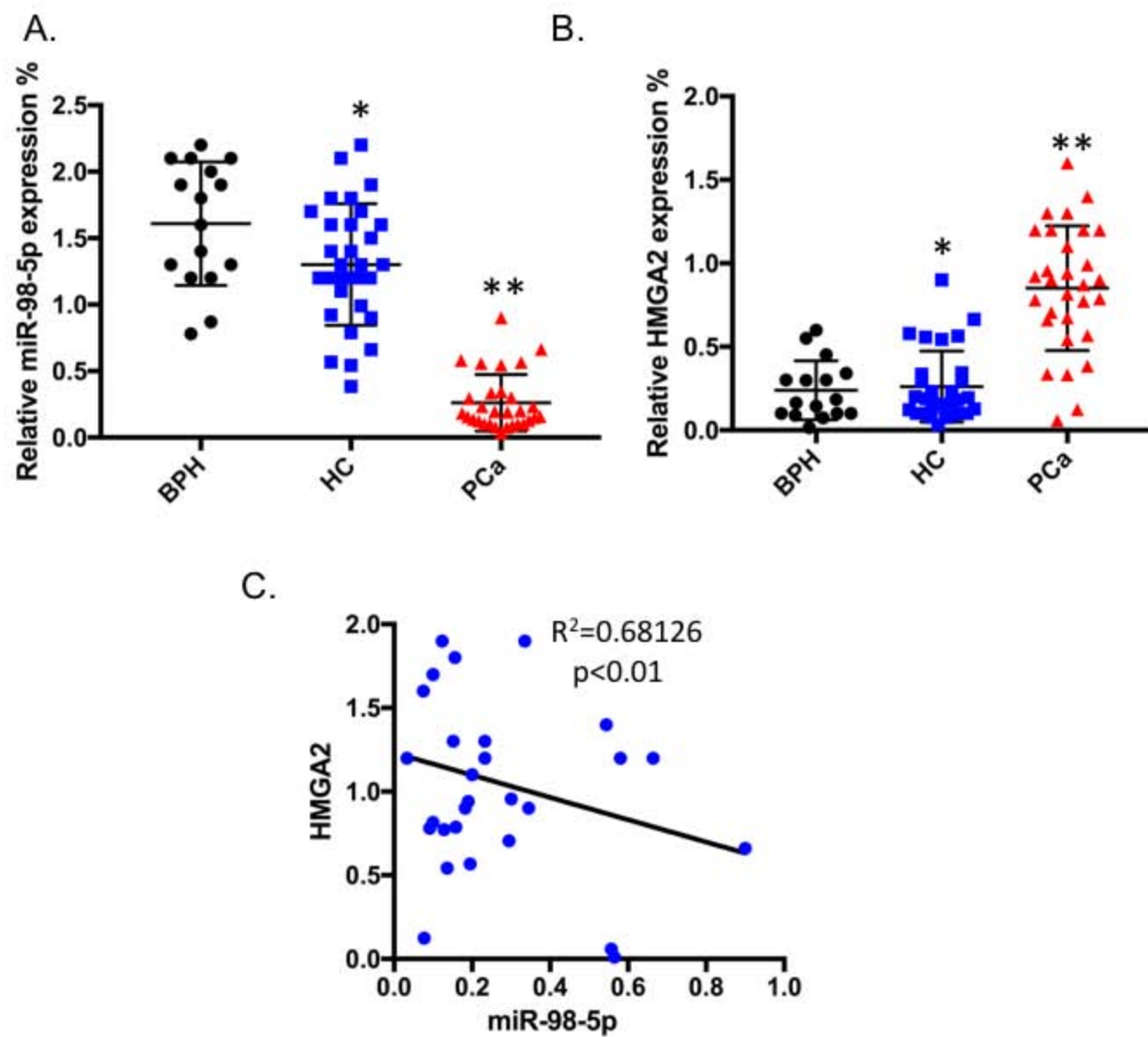

Supplement: Supplementary file 1 [file bsr20190635_Supp1.pdf]
